# Microstructural Evolution during DPRM Process of Semisolid Ledeburitic D2 Tool Steel

**DOI:** 10.1155/2013/828926

**Published:** 2013-10-02

**Authors:** M. N. Mohammed, M. Z. Omar, J. Syarif, Z. Sajuri, M. S. Salleh, K. S. Alhawari

**Affiliations:** Department of Mechanical and Materials Engineering, Faculty of Engineering and Built Environment, Universiti Kebangsaan Malaysia, 43600 Selangor, Malaysia

## Abstract

Semisolid metal processing is a relatively new technology that offers several advantages over liquid processing and solid processing because of the unique behaviour and characteristic microstructure of metals in this state. With the aim of finding a minimum process chain for the manufacture of high-quality production at minimal cost for forming, the microstructural evolution of the ledeburitic AISI D2 tool steel in the semisolid state was studied experimentally. The potential of the direct partial remelting (DPRM) process for the production of AISI D2 with a uniform globular microstructure was revealed. The liquid fraction was determined using differential scanning calorimetry. The microstructures of the samples were investigated using an optical microscope and a scanning electron microscope equipped with an energy dispersive spectroscopy analyser, while X-ray phase analysis was performed to identify the phase evolution and the type of carbides. Mechanical characterisation was completed by hardness measurements. The typical microstructure after DPRM consists of metastable austenite which was located particularly in the globular grains (average grain size about 50 **μ**m), while the remaining interspaces were filled by precipitated eutectic carbides on the grain boundaries and lamellar network.

## 1. Introduction

AISI D2 is the most commonly used high-carbon and high-chromium cold-work tool steel. It has many attractive properties such as high wear resistance, high compressive strength, and high hardness after hardening which can be an advantage for a variety of industrial applications such as mill rolls, blanking dies, and punches. However, the conventional routes (casting and forging) consume a lot of energy and time. Consequently, an innovative route that involves less energy consumption and shorter processing time and can substitute the conventional manufacturing routes is desired.

Following on the work of Spencer et al., which identified the essential thixotropic properties [1], semisolid metal (SSM) processing received considerable attention around the world owing to its ability to facilitate high-quality production at a relatively lower cost when compared with conventional processes. For instance, compared to casting, the quality of the final product can be improved because there are fewer defects related to the solidification processes. Also, compared to the forging processes, the required forming loads are much lower, and thus complex shaped parts can be produced net or near-net in a one-step process [2, 3]. A key step in the successful operation of SSM processing is the appropriate production of feedstock with a thixotropic property. In general, technologies for SSM processing can be divided into two main categories based on the status of the starting material: either a liquid-phase or a solid-phase alloy [4, 5]. Currently, there are a number of methods that can be used to achieve a globular microstructure, such as cooling slope, mechanical stirring, magnetohydrodynamic stirring (MHD), and direct partial remelting (DPRM) [6–8].

The direct partial remelting method (DPRM) is considered one of the most effective and commercially viable solid state processes (especially high-melting-point metals) to produce a nondendritic microstructure in order to gain some insight into the microstructural development of the starting material when in the semisolid state. The partial remelting experiments can be directly reheated to its semisolid range from the as-received state without having to go through the conventional feedstock preparation routes, like Stress-Induced and Melt-Activated (SIMA), Recrystallization and Partial Melting (RAP), and various others [8]. This indicates a widening of the range of potential routes to thixoformable microstructures. Furthermore, Zinc-based alloys are among the potential candidates for semisolid processing which offers significant advantages, such as reduction of macrosegregation, porosity, and low forming efforts [9]. The advantage of semisolid metal (SSM) over conventional process is that it can utilize the unique thixotropic behaviour which can be found in the semisolid state. The majority of the research effort in respect of SSM processing techniques is focused on low-melting-point alloys such as aluminium and magnesium alloys. There is also extensive interest in semisolid processing higher temperature materials such as copper-based alloys, cast iron, steels, and composites [10, 11]. There are only a few studies that have been undertaken on the development of high-melting-point materials for the thixoforming process [12]. However, the trend of researchers is becoming increasingly interested in this goal due to the advantages of steel thixoforming [13]. An incredibly promising candidate for successful semisolid material application is high-carbon, high-chromium ledeburitic tool steel. Due to the growing demand for cold-work tool steel in various industrial applications, it is crucial to improve the fabrication technique because complex shapes involve an extensive and costly workshop effort. Hence, a one-step net-shaping process, such as semisolid forming, could offer great benefits. This study was employed to characterize the microstructural spheroidisation and phase transformations of AISI D2 cold-work tool steel at the isothermal temperature during partial remelting method.

## 2. Experimental Materials and Procedure

**Figure 1:**
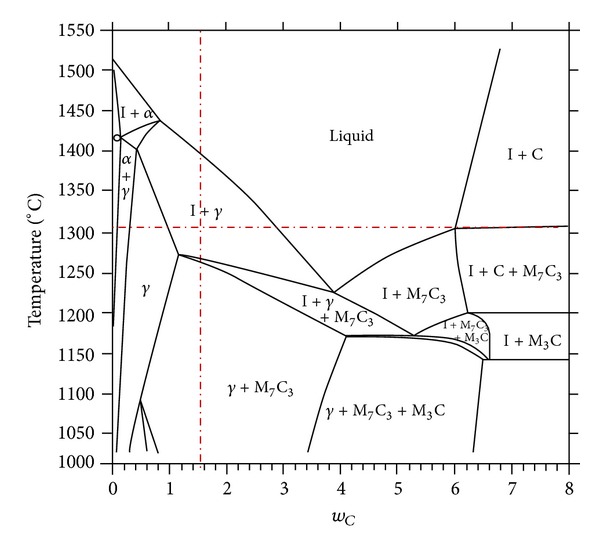
Binary phase diagram of AISI D2 tool steel with variable carbon content [14].

The material used in this work is AISI D2 cold-work tool steel which was supplied after a soft annealing process, that is, heating to 850°C followed by cooling at 10°C/h to 650°C and finally air cooling. The chemical composition of the starting material determined by X-ray fluorescence (XRF) technique is shown in Table 1.  Figure 1 displays the part of the phase diagram for AISI D2 tool steel with variable carbon content. The dotted line shows the carbon content corresponding to the investigated steel.

Differential scanning calorimetry (DSC) analysis was carried out primarily to estimate the solidus and liquidus temperatures and the liquid fractions within the semisolid transition range. The as-received material was cut into small pieces (less than 20 mg) for testing by using a Netzsch-STA (TG-DSC) 449 F3 simultaneous thermogravimeter. The heating rate employed was 10°C/min in a nitrogen atmosphere to prevent oxidation. Determination of the liquid fraction was achieved through analysis of the partial integral of the area under the curve of the endothermic. Transformations of phases are detected on the DSC heating curve as deviations because of the energy release or consumption. The magnitude of deviation is correlated to the phase's quantity. The temperatures of each peak were defined, and the changes proportionate to certain peaks were verified.

**Figure 2:**
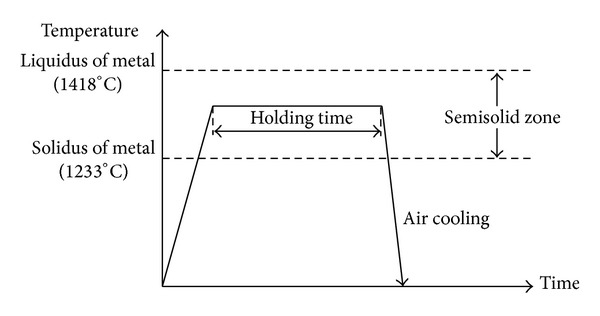
Schematic of DPRM experiment.

The DPRM experiment was performed using a vertical, high-temperature carbolite furnace with a protective atmosphere (argon gas). The material was cut into samples with a size of Φ16 × 100 mm. When the furnace had reached the predefined temperature, the sample was lowered into the hottest place inside the furnace by using a chromel wire to ensure rapid heating of the sample. The selected temperature for this experiment was selected based on the DSC test. After the desired temperature was reached, the sample was held for 5 minutes and then subjected to air cooling to room temperature. The experimental process is illustrated in Figure 2.

Microstructural characterisation was carried out using a BX-51 Olympus optical microscope and a Hitachi S3400N scanning electron microscope (SEM), equipped with energy dispersive spectroscopy (EDS). X-ray phase analysis was performed to identify the phase evolution and the type of carbides. Average grain sizes were calculated by using the mean lineal intercept method (after the ASTM E112-96 standard) and the shape factor equation *F* = *U* ^2^/4*πA*, where *F* is the shape factor, *U* is the grain circumference, and *A* is the grain area. When *F* equals 1, the grains are completely globule, but when *F* is greater than 1, a more complex shape can be obtained [15]. According to Hirt et al., the ideal grain size for semisolid forming should not exceed 100 *μ*m, and the globularity (shape factor) must not be greater than 2 [16]. All samples were etched using Villela reagent (1 g picric acid, 5 mL hydrochloric acid, and 95 mL ethyl alcohol) to reveal their microstructures. Mechanical characterisation was completed by Vickers hardness (HV) using a load of 2 kg applied for 15 seconds.

## 3. Results and Discussion

### 3.1. Starting Material

**Figure 3:**
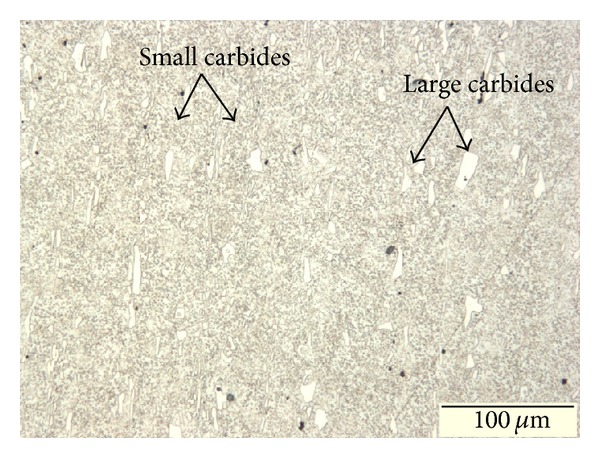
Optical micrographs of as-received AISI D2.

**Figure 4:**
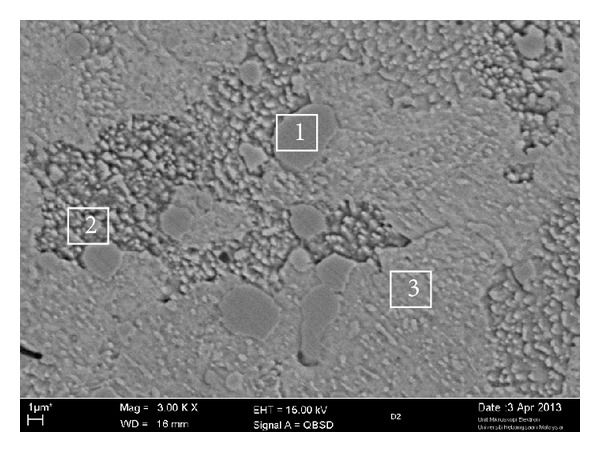
SEM micrograph of as-received AISI D2.

The as-received material contains surplus arrays of coarse large carbides with smaller carbides distributed homogeneously in a ferrite matrix parallel to the working direction, as shown in Figure 3. This structure has been identified in annealed cold-work tool steel and thus confirms that soft annealing treatment has been carried out as described by the material supplier [17]. The initial soft-annealed microstructure of the samples was observed with the SEM and was found to consist of carbides in a ferrite matrix, as shown in Figure 4. The chemical compositions of the analysed carbides and the ferrite matrix are given in Table 2. It can be seen that the M_7_C_3_ carbides are composed mainly of C, V, and Cr. The absence of MC carbide in the as-received AISI D2 tool steel can be explained by the soft annealing process at 850°C due to dissolution temperature of MC carbide at 740°C. The amount of M_23_C_6_ carbide after cooling to room temperature is negligible as compared to M_7_C_3_ carbides [18].

### 3.2. Liquid Fraction Profile and Transformations of Phases

**Figure 5:**
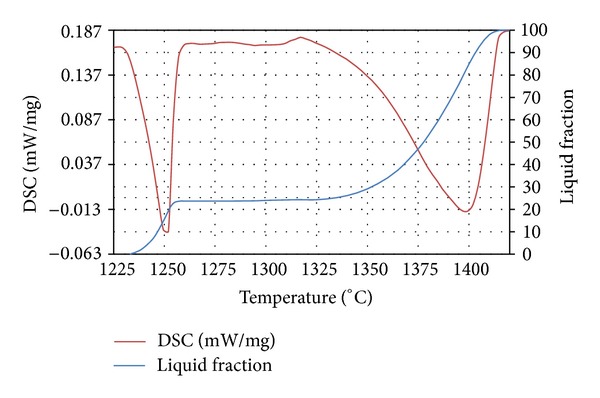
DSC heating flow and liquid fraction curves for AISI D2.

The melting point is defined as the beginning of the endothermic peak. The onset of melting is set at such a temperature that the heat curve drops away from the tangent line. The end of melting is set by the beginning point on the other side of the peak. From Figure 5, it can be seen that the melting interval consists of two peaks; the first one is connected to the carbide dissolution and the second one corresponds to the final melting of austenite. The endothermic effect starts at 1233°C and ends at 1418°C. Phase transformations in the AISI D2 were analysed by using the TGA-DSC instrument mentioned above. In general, thixoforming is performed at a liquid fraction between 20% and 50% [17]; consequently, when the AISI D2 tool steel is partially remelted at 1300°C, the corresponding liquid fraction is approximately 25%.

### 3.3. Semisolid State Morphology and Evolution

**Figure 6:**
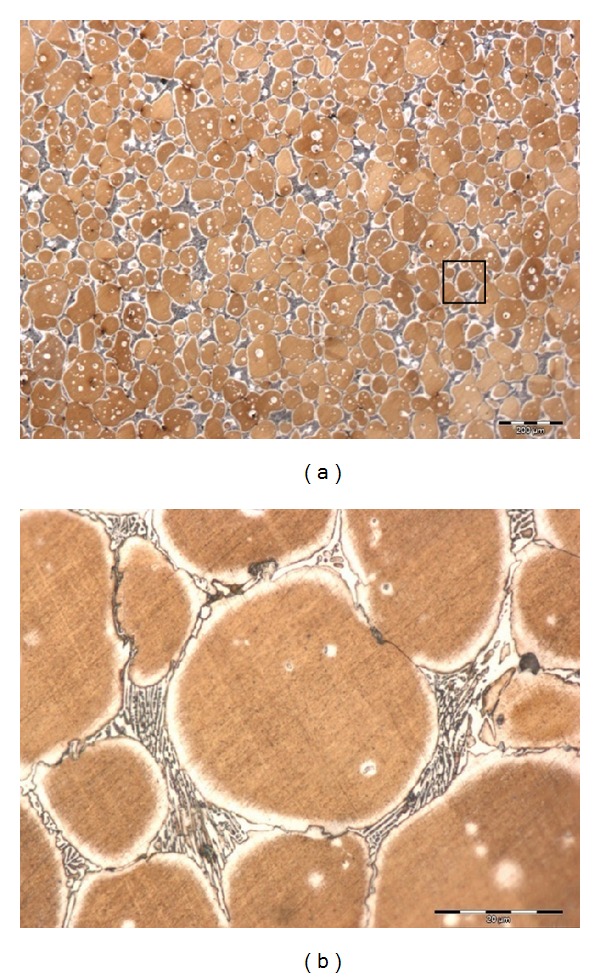
Microstructural development of AISI D2 after DPRM at 1300°C with a holding time of 5 minutes; (b) is the area within the black rectangle shown in (a).

For the AISI D2 tool steel, experiments were implemented in the corresponding temperature range, and the microstructure was dissected by means of classic metallography. A fine austenitic spherical primary phase was obtained after the reheating treatment of the as-received material at temperatures of around 1300°C, as shown in Figure 6. A spherical shape of this type is one of the most important basic requirements for the thixoforming application. Microstructural observation revealed that there was a good arrangement of spherical grains with a grain size of 50 *μ*m and shape factor of 1.16, which are surrounded by an eutectic mixture. It can be clearly seen that the grains are separated from each other because of a thick liquid phase between the grain boundaries. If enough wetting is achieved along the grain boundaries, initial shear during forming may cause the grains to flow past each other and the semisolid slurry will flow thixotropically.

In this case, the morphologies and compositions of AISI D2 after treatment, seen in Figure 6, differ from those seen in the as-received samples. The transformation into globular grains is supported by the carbide dissolution process. The dissolution of different types of carbides is beneficial in that it gives a big semisolid interval and a little temperature sensibility [17]. However, in respect of the microstructure and temperature, it can be seen that with increasing isothermal temperature, the liquid phase starts to diffuse and distribute into grain boundaries and spread more evenly to make a solid-phase globular structure with favourable grain size for thixoforming.

**Figure 7:**
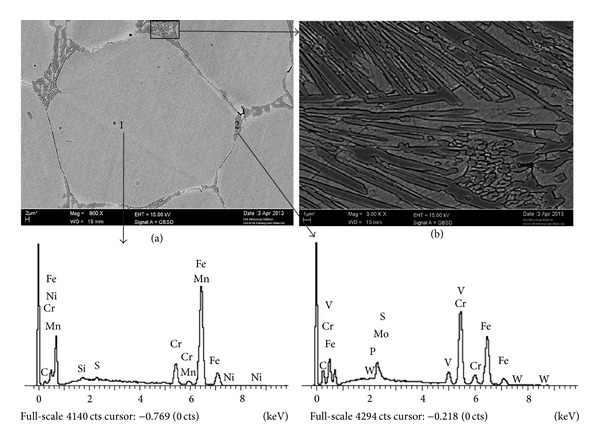
SEM micrographs of AISI D2 sample after being partial remelted at 1300°C with EDX analysis at points 1 and 2. Note that (b) is the area within the black rectangle shown in (a).

Scanning electron microscopy and EDX analysis combined with EDS analysis were carried out in order to scrutinise the phases that occurred during the DPRM process.  Figure 7 illustrates the EDX analysis results from the SEM image taken from points 1 and 2. From point 1, the EDS results confirm that metastable austenite was present, particularly in the globular grains. The remaining interspaces were filled by precipitated eutectic carbides on the grain boundaries and on the lamellar network which is indicated by the increased content of chromium (Cr), vanadium (V), and carbon (C) in the eutectic area, as shown at point 2.

**Figure 8:**
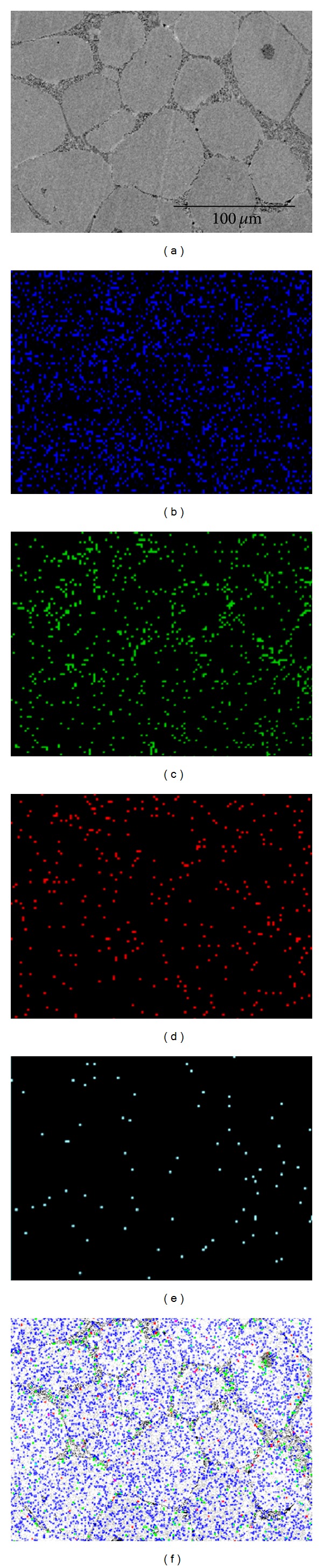
SEM-EDX elemental mapping of AISI D2, showing (a) SEM image, (b) Fe, (c) Cr, (d) C, (e) V, and (f) overall.

For the more detailed explanation of the microstructure, SEM-EDX elemental mapping techniques were used. On the base of element distribution, it can be deduced that Fe localized particularly in the globular grains while an increased content of V, C, and Cr, which is distributed uniformly on the boundaries of the globular particles (eutectic area), denotes high content of carbides, as shown in Figure 8.

### 3.4. XRD Analysis

**Figure 9:**
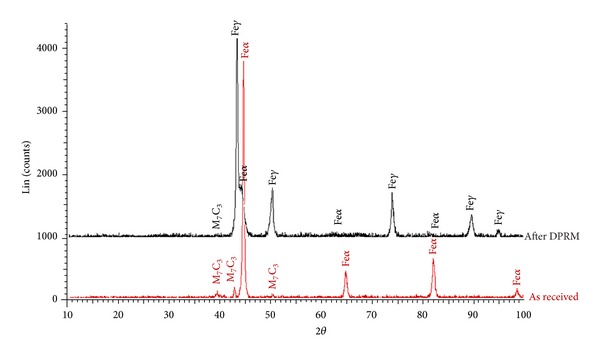
X-ray diffraction pattern for as-received and after direct partial remelting (DPRM).

X-ray diffraction (XRD) analysis was performed in order to identify the phase evolution and the type of carbides formed after direct partial remelting. The X-ray diffraction patterns for as-received tool steel and after heat treatment are given in Figure 9. The analysis of the diffraction patterns of as-received tool steel illustrates peaks corresponding to ferrite phase and iron-chromium carbide (M_7_C_3_). The XRD pattern of the tool steel after DPRM reflects the main three phases: austenite, ferrite, and carbide M_7_C_3_ in the matrix. The presence of austenite is primarily connected with the increased content of Cr and C in the solid solution stabilising the austenite. Higher contents of these elements have a significant effect on the martensite temperature, and consequently the transformation of austenite to martensite is very difficult. In addition, when quick cooling from the solid-liquid range is utilised, there is relatively better stability for the austenitic phase at room temperature.

### 3.5. Hardness Properties

The hardness of the material after DPRM was tested and found to be about 350 Hv, which is higher than the hardness of the as-received tool steel material, which was 220 Hv. This indicates phase transformation in which ferrite and carbides change into metastable austenite [19]. These structures are especially attractive due to their mechanical properties being more related to the precipitated carbides and the dissolved alloying elements in ledeburitic steel.

## 4. Conclusion

The work presented herein proposed new prospects for the semisolid processing of steels. The current study shows that the microstructure was composed of metastable austenite which was located particularly in the globular grains, while the remaining interspaces were filled by precipitated eutectic carbides on the grain boundaries and lamellar network. The crystal of the globular structure was specified as austenite by using SEM-EDX elemental mapping and confirmed by XRD analysis, while the eutectic contains ferrite with fine M_7_C_3_ carbide particles. High contents of chromium and carbon in the solid solution combined with rapid solidification from the solid-liquid range were observed, which indicated relatively better stabilisation for the austenitic phase at room temperature. Based on the requirements of thixoformability, the work confirms the suitability of the AISI D2 cold work tool steel as a candidate material for semisolid processing.

## Figures and Tables

**Table 1:** Chemical composition of AISI D2 (wt.%) cold-work tool steel by XRF technology.

C	Si	Mn	P	S	Cr	Ni	Mo	V	W	Cu	Fe
1.55	0.258	0.239	0.025	0.01	11.2	0.197	0.79	0.85	0.2	0.08	Balance

**Table 2:** EDX analysis results for the carbides and ferrite matrix wt.% shown in Figure 4.

	C	Si	P	S	V	Cr	Mn	Fe	Ni	Mo	W	Phase
Point 1	6.03	0.1	0.08	0.03	5.07	45.95	0.34	40.58	0.07	1.46	0.29	M_7_C_3_
Point 2	6.70	0.16	0.02	0.24	5.52	44.51	0.49	40.62	0.09	1.57	0.10	M_7_C_3_
Point 3	1.61	0.27	0.02	0.02	0.3	9.2	0.35	87.20	0.29	0.29	0.45	*α*
